# All it takes is empathy: how virtual reality perspective-taking influences intergroup attitudes and stereotypes

**DOI:** 10.3389/fpsyg.2023.1265284

**Published:** 2023-09-18

**Authors:** Vivian Hsueh Hua Chen, Gabrielle C. Ibasco

**Affiliations:** ^1^Wee Kim Wee School of Communication and Information, Nanyang Technological University, Singapore, Singapore; ^2^Department of Media and Communication, Erasmus University Rotterdam, Rotterdam, Netherlands; ^3^Department of Psychology, The University of British Columbia, Vancouver, BC, Canada

**Keywords:** perspective-taking, intergroup attitudes, empathy, stereotypes, virtual reality

## Abstract

Research in the past decade has demonstrated the potential of virtual reality perspective-taking (VRPT) to reduce bias against salient outgroups. In the perspective-taking literature, both affective and cognitive mechanisms have been theorized and identified as plausible pathways to prejudice reduction. Few studies have systematically compared affective and cognitive mediators, especially in relation to virtual reality, a medium posited to produce visceral, affective experiences. The present study seeks to extend current research on VRPT’s mechanisms by comparing empathy (affective) and situational attributions (cognitive) as dual mediators influencing intergroup attitudes (affective) and stereotypes (cognitive). In a between-subjects experiment, 84 participants were randomly assigned to embody a VR ingroup or outgroup waiting staff at a local food establishment, interacting with an impolite ingroup customer. Results indicated that participants in the outgroup VRPT condition reported significantly more positive attitudes and stereotypes towards outgroup members than those in the ingroup VRPT condition. For both attitudes and stereotypes, empathy significantly mediated the effect of VRPT, but situational attributions did not. Findings from this research provide support for affect as a key component of virtual experiences and how they shape intergroup perceptions. Implications and directions for further research are discussed.

## 1. Introduction

Virtual reality (VR) has often been touted as an “empathy machine” (Bevan et al., 2019; Barbot and Kaufman, 2020; Hassan, 2020). Through avatar embodiment with visuo-motor synchrony in VR, the user perceives themselves as inhabiting the body of someone else, regardless of actual differences between the user and avatar (Yee and Bailenson, 2007; Tham et al., 2018). This process enables virtual reality perspective-taking (VRPT) (Herrera et al., 2018; Loon et al., 2018; Mado et al., 2021). VRPT of an avatar with a different salient identity from the player can lead to significant improvements in attitudes toward these identities—including Black people (Peck et al., 2013; Banakou et al., 2016; Behm-Morawitz et al., 2016), women (Lopez et al., 2019), the homeless (Herrera et al., 2018), and immigrants (Chen et al., 2021c). However, some studies found no effect on reducing bias against outgroups (Groom et al., 2009; Hasler et al., 2017). One reason for these inconsistent findings may be a limited understanding of the specific psychological mechanisms underlying the effects of VRPT, and how to best optimize these mechanisms for positive intergroup outcomes.

Theoretically, perspective-taking has both cognitive and affective mechanisms (Todd and Galinsky, 2014). Empirical studies comparing affective and cognitive mechanisms are somewhat inconsistent and have only been conducted in a non-VR context. Cognitively, perspective-taking has been found to subvert general attributional biases for behavior (Regan and Totten, 1975; Vescio et al., 2003; Hooper et al., 2015). In the intergroup context, people tend to attribute undesirable actions made by their ingroup to unstable, external factors and those made by an outgroup according to stable, internal traits (Pettigrew, 1979). Subverting this tendency, perspective-taking can encourage people to attend to context-specific, situational factors shaping outgroup behavior as they would do for their own ingroup (Todd et al., 2012). In a series of studies, Vescio et al. (2003) experimentally induced the non-VR perspective-taking of stigmatized group members and found that an increase in situational attributions for outgroup behavior positively mediated the effect of perspective-taking on affective attitudes toward the outgroup. To our knowledge, only one study has tested the role of behavioral attributions in the context of VRPT. Contrary to results found for non-VR perspective-taking, in this study, attributions did not explain the effect of VRPT on perceptions of a racial minority group (Chen et al., 2021b).

On the other hand, an affective mechanism, empathy, has been studied extensively as a link between perspective-taking and intergroup bias. Empathy has been identified as an outcome of both VRPT and non-VR perspective-taking (Decety, 2007; Shih et al., 2013; Herrera et al., 2018; Boehm, 2020). In a longitudinal study, Herrera et al. (2018) discovered that the VRPT of a homeless person induced greater empathy, more durable positive attitudes over time, and greater willingness to sign a petition supporting the homeless when compared against a less immersive non-VR perspective-taking manipulation. This study did not test the role of empathy as a mediator, however. Other studies found empathy to indeed mediate the effect of non-VR perspective-taking on intergroup perceptions (Batson et al., 1997; Vescio et al., 2003; Shih et al., 2009). However, Todd and Burgmer (2013) found that empathy did not mediate the effect of non-VR perspective-taking on implicit attitudes, although perspective-taking did lead to an overall increase in empathic arousal toward the outgroup.

One reason for these divergent findings on empathy and attributions may be how studies manipulate perspective-taking. Non-VR studies that found a mediating effect of empathy (e.g., Batson et al., 1997; Shih et al., 2009) asked participants to focus on how a member of stigmatized outgroup might be thinking or feeling, before presenting them with a narrative about this outgroup member (Batson et al., 1997). In contrast, Todd and Burgmer’s (2013) manipulation did not provide relevant information about an outgroup member’s hardships or struggles on the basis of their identity. The absence of information that typically warrants concern or compassion in this study may have weakened the inducement of empathy.

Furthermore, only one study thus far has directly compared empathy and attribution style as mediators for the same non-VR perspective-taking manipulation (Vescio et al., 2003). Vescio and colleagues found both mediators to be significant, and attributions had a stronger and more consistent mediating effect than empathy. This comparison, however, was primarily based on an assessment of which indirect effect size appeared larger, rather than a pairwise contrast analysis to ascertain whether one indirect effect was greater than another to a degree of statistical significance (Preacher and Hayes, 2008; Hayes, 2017). To the best of our knowledge, no studies have tested VRPT’s influence on cognitive attribution style, nor statistically compared empathy and attribution style as mediators simultaneously in the relationship between VRPT and intergroup perceptions.

The present study aims to apply Vescio and colleagues’ theoretical approach in a VRPT context by examining empathy and attribution style as dual mediators of the effect of VRPT on two commonly used measures of intergroup prejudice—attitudes (affective) and stereotypes (cognitive)—toward an immigrant outgroup. For parsimony, we use the term “attitudes” to refer to *affective* attitudes (i.e., feelings directed toward an outgroup) specifically throughout this paper, acknowledging that “attitudes” has been used to refer to both affective and behavioral aspects in past work.

Although other affective and cognitive mechanisms (e.g., self-other overlap) may contribute to the effects of VRPT, we focus on empathy and situational attributions primarily due to the precedent set by Vescio and colleagues, who did the same in a non-VR context, as well as our aim to test mechanisms that are sufficiently distinct from one another. There is some debate about whether self-other overlap, for example, closely overlaps with and may not be entirely distinct from certain facets of empathy (Preston and Hofelich, 2012). As such, comparing empathy with self-other overlap (instead of attribution style) as mediators may be less informative due to their relatively high amount of shared variance.

Aligning with past research, participants embodied a VR character, enabling perspective-taking of an outgroup immigrant who experiences a microaggression in their workplace. In the control condition, participants take the perspective of an ingroup character in the same scenario. We propose the following hypotheses:

*H1*: VRPT of an outgroup character will lead to significantly more positive attitudes and stereotypes than VRPT of an ingroup character.

*H2*: Affective empathy positively mediates the effect of VRPT of an outgroup character on attitudes and stereotypes.

Due to the lack of empirical evidence supporting the mediating role of situational attributions in the VRPT literature, we propose the following as research questions instead of hypotheses:

*RQ1*: Do situational attributions mediate the effect of VRPT of an outgroup character on attitudes and stereotypes?

*RQ2*: Are there significant differences between the indirect effects of outgroup VRPT via empathy and situational attributions on attitudes and stereotypes?

## 2. Materials and methods

### 2.1. Participants

A total of 84 Singaporean participants, with an age range of 19 to 33 years old, were recruited from a public autonomous research university in Singapore. The sample allows for 80% power to detect a both a significant medium-sized positive experimental effect (*f* = 0.22) and correlation (*r* = 0.27). Recruitment emails for participation were send out to a random selection of 15 student email lists provided by the university. All participants identified as being Singaporean Chinese, of whom 51 were female (60.7%) and 33 were male (33%). Participants were compensated with either course credit (for students) or SGD $10 gift cards. Prior to recruitment, this study received ethics approval from the Institutional Review Board of (university suppressed).

### 2.2. Design and procedure

This study featured a one-way between-subjects experimental design, in which participants were randomly assigned to take the perspective of either a Chinese immigrant from the People’s Republic of China (PRC; outgroup VRPT, *n* = 46) or a Singaporean Chinese citizen (ingroup VRPT, *n* = 38) in VR. Recent studies have found that Chinese immigrants from PRC face significant prejudice in Singapore, and compared to other immigrant groups (e.g., Indians, Americans), are typically stereotyped to be the least warm and poses the greatest symbolic threat (Ramsay and Pang, 2017; Chen et al., 2021a). Despite sharing ethnic lineage with the Chinese-majority demographic of Singapore, PRC Chinese are often viewed to have a less modern culture than Singaporeans and do not speak English well (Ang, 2018).

The VR environment used was a lunchtime food court simulation. The participant embodied a waiting staff with the first-person viewpoint, using an HTC Vive headset. The interaction in VR environment is done through voiceover and in English. A voiceover narration, recorded by a native North American speaker, guided participants through 3 scenes. The English accents of the embodied VR characters and NPCs were matched with their nationalities and were different from the narrator’s accent. The first scene aimed to enhance embodiment. Participants started in a room facing a mirror to establish familiarity with their avatar and visuo-motor synchrony. They were able to see their face and body while interacting with the VR environment. Participants were instructed to interact with their character’s belongings in front of them, which included a Singaporean citizen’s identity card in the ingroup VRPT condition, or a foreign worker’s identity card in the outgroup VRPT condition.

In the second scene, participants were tasked with serving incoming customers by taking orders and interacting with ingredients placed on the cooking counter. The food court was crowded and the various customer NPCs’ ethnicities reflected a typical lunchtime crowd in Singapore. In both conditions, the narrator prefaced that the participant’s character is fluent in Mandarin Chinese, but may struggle to understand complex orders in English. Participants then encountered one irritable non-playable character (NPC) customer, whose order their character failed to understand initially. The NPC customer was a Singaporean Chinese female and her voice exhibited frustration toward the participant in both conditions.

In the final scene, while tasked with cleaning tables, participants received a phone call from their child—who either had a PRC Chinese accent (outgroup VRPT condition) or a Singaporean Chinese accent (ingroup VRPT condition)—requesting money for a school trip. This scene was designed to highlight the struggles faced by the participant’s character in financing their child’s education.

### 2.3. Measures

Participants completed a pre-VR and post-VR questionnaire that captured their stereotypes and attitudes toward the immigrant outgroup (PRC Chinese). Attributions and empathy were only measured in the post-VR questionnaire.

#### 2.3.1. Attitudes

Attitudes were measured both pre- and post-VR using an affective feeling thermometer scale (Alwin, 1997). Participants were asked to “indicate their attitudes towards PRC Chinese” across three dimensions: “cold (1)…warm (100),” “unfavorable (1)…favorable (100),” and “negative (1)…positive (100).” A higher score on the scale indicates more positive attitudes toward the outgroup (pre-VR: *M* = 56.08, SD = 20.99, Cronbach’s alpha = 0.92; post-VR: *M* = 61.33, SD = 20.54, Cronbach’s alpha = 0.95).

#### 2.3.2. Stereotypes

A series of semantic differential items (Osgood et al., 1975) measured the valence of participants’ stereotypical beliefs about the outgroup in both pre- and post-VR questionnaires. Each item presented two opposing adjectives, on each side of a 7-point Likert-type scale. Items included “rude (1)…polite (7)” and “dishonest (1)…honest (7).” A higher score on the scale indicates less negative stereotyping (pre-VR: *M* = 3.79, SD = 0.99, Cronbach’s alpha = 0.87; post-VR: *M* = 4.18, SD = 0.98, Cronbach’s alpha = 0.90).

#### 2.3.3. Situational attributions

In the post-VR questionnaire, a series of bipolar scale items adapted from Miller et al. (1981) asked participants to rate how natural it is for PRC Chinese to engage in negative or undesirable behaviors on a 7-point Likert-type scale. Items included “rude by nature (1)…rude only when the situation calls for it (7)” and “inherently quarrelsome (1)…quarrelsome only when they have to be.” A higher score on this scale suggests more situational, versus dispositional, attributions made about negative outgroup behavior (*M* = 5.93, SD = 1.03, Cronbach’s alpha = 0.82).

#### 2.3.4. Affective empathy

In the post-VR questionnaire, participants indicated their level of agreement with three items adapted from Davis (1980) interpersonal reactivity index. These items assessed affective empathy toward the outgroup following VRPT, including items such as “I felt compassion for the Chinese PRC,” “I felt sorry for the Chinese PRC,” and “I felt protective towards the Chinese PRC” (*M* = 4.52, SD = 1.74, Cronbach’s alpha = 0.91).

#### 2.3.5. Manipulation check

To ensure that participants were cognizant of which characters they played in the VR scenario, the post-VR questionnaire asked participants to report the correct group identity of the character they embodied—the Singaporean Chinese, or the PRC Chinese. Those in the ingroup VRPT condition were significantly more likely than those in the outgroup VRPT condition to correctly report embodying the ingroup character, *X*^2^ (1, 84) = 80.05, *p* = 0.000.

Correlations between all variables are presented in Table 1.

**Table 1:** Descriptive statistics, reliability, and correlations between all measured variables.

	*M*	SD	*α*	1	2	3	4	5	6
**Pre-VR**									
1. Attitudes	56.09	20.99	0.92	1					
2. Stereotypes	3.79	0.99	0.87	0.58^**^	1				
**Post-VR**									
3. Attitudes	61.33	20.54	0.95	0.79^**^	0.51^**^	1			
4. Stereotypes	4.18	0.98	0.90	0.42^*^	0.67^**^	0.63^**^	1		
5. Empathy	4.52	1.74	0.91	0.19	0.14	0.42^**^	0.38^**^	1	
6. Attributions	5.93	1.03	0.82	0.35^**^	0.54^**^	0.39^**^	0.59^**^	0.23^*^	1

M, mean; SD, standard deviation; *α*, Cronbach’s alpha. ^*^p < 0.05; ^**^p < 0.01.

## 3. Results

Two between-subjects ANCOVA tests with a Bonferroni post-hoc correction were conducted to assess the effect of outgroup embodiment on both attitudes and stereotypes (taken from the post-VR questionnaire), controlling for baseline measures (recorded in the pre-VR questionnaire). Conventions in pre-post experimental designs suggest that an ANCOVA testing experimental effects on post-test measures, while adjusting for pre-test measures, typically leads to the most unbiased estimates when compared to alternative approaches (e.g., ANOVA on just post-test measures, repeated-measures ANOVA to estimate an effect on changes from pre- to post-test (O’Connell et al., 2017)). An ANCOVA on post-test scores also affords greater power in randomized experimental studies than a repeated-measures ANOVA assessing a change in scores over time (Van Breukelen, 2006). By controlling for pre-VR baseline scores, we are able to rule out the possibility that observed differences of our VRPT manipulation are due to baseline differences in prejudice.

As predicted by H1, VRPT of an outgroup led to both significantly more positive feeling thermometer scores, *F*(1, 81) = 7.89, *M*_diff_ = 7.58, SE = 2.67, *p* = 0.006, (2.21, 12.96), *η*_p_^2^ = 0.09, and stereotypes directed towards the outgroup, *F*(1, 81) = 8.12, *M*_diff_ = 0.45, SE = 0.16, *p* = 0.006, (0.13, 0.76), *η*_p_^2^ = 0.09.

Prior to conducting our primary mediation model, we first tested whether the VRPT manipulation had significant effects on empathy and situational attributions in separate *t*-tests. Compared to participants in the control condition, participants exposed to the VRPT manipulation reported higher levels of empathy for the PRC Chinese, *t*(82) = 7.30, *M*_diff_ = 2.20, SE = 0.30, *p* = 0.000, but did not make significantly more situational attributions for negative PRC Chinese behavior, *t*(82) = 0.16, *M*_diff_ = 0.04, SE = 0.23, *p* = 0.87.

To test our main hypotheses and research questions, we conducted parallel mediation analyses using PROCESS (Hayes, 2017) Model 4 on SPSS v24 with 5,000 bootstraps to compare how affective empathy and situational attributions mediate the effect of VRPT on both attitudes and stereotypes. Baseline scores for each dependent variable were set as covariates to examine the effect of the VRPT manipulation and our proposed mediators independent of baseline prejudice. To assess the statistical difference between two parallel mediators, we utilized (Hayes, 2017) normal theory approach that constructs a bootstrapped 95% confidence interval around the estimated difference between two indirect effects.

**Figure 1:**
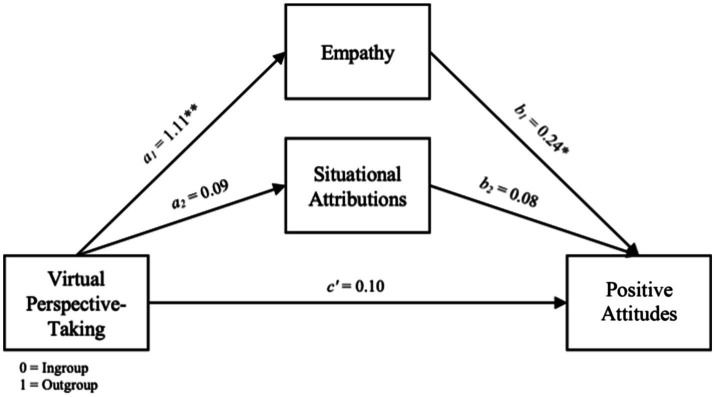
Parallel mediation model on positive attitudes toward the outgroup.

The overall model predicting feeling thermometer scores was significant, *F*(4, 79) = 44.90, *R*^2^ = 0.69, *p* = 0.000. The total effect of VRPT was significant, *b* = 7.58, SE = 2.70, *t*(82) = 2.81, *p* = 0.006, (2.21, 12.96), and this effect was primarily driven by the indirect effects in the model, as the direct effect of the manipulation was not significant, *b* = 1.18, SE = 3.38, *t*(82) = 0.35, *p* = 0.73, (7.92, 0.58). Supporting H2, empathy produced a significant positive indirect effect, as the bootstrapped confidence interval included zero, *b* = 5.47, SE = 2.31, (1.20, 10.35), but the same effect was not found for situational attributions, *b* = 0.16, SE = 0.44, (−0.66, 1.13) (see Figure 1). A contrast analysis also revealed that empathy had a significantly greater indirect effect on feeling thermometer scores than situational attributions, *b*_diff_ = 0.26, SE = 0.11, (0.05, 0.49).

**Figure 2:**
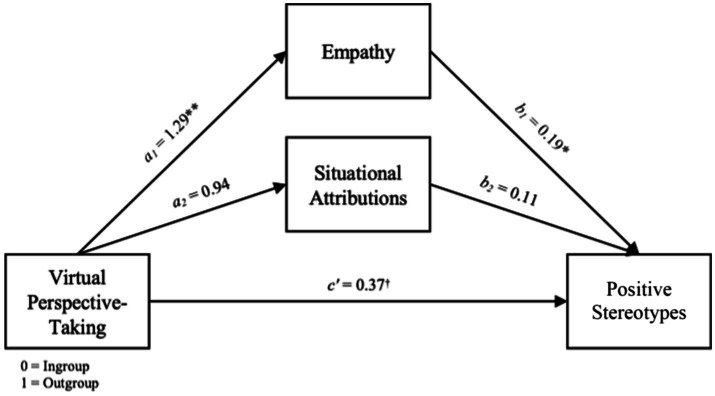
Parallel mediation model on positive stereotypes toward the outgroup.

The model predicting semantic-differential stereotypes was also significant, *F*(4, 79) = 26.03, *R*^2^ = 0.57, *p* = 0.000. The total effect of outgroup VRPT was significant, *b* = 0.45, SE = 0.16, t = 2.85, *p* = 0.006, (0.13, 0.76), including a non-significant direct effect, *b* = 0.18, SE = 0.19, *t*(82) = 0.98, *p* = 0.329, (−0.19, 0.56) and a significant positive indirect effect of empathy, *b* = 0.24, SE = 0.11, (0.02, 0.47), supporting H2. The indirect effect of situational attributions was not significant, *b* = 0.02, SE = 0.06, (−0.07, 0.15) (see Figure 2), and empathy produced a significantly greater indirect effect on stereotypes than attributions, *b*_diff_ = 0.21, SE = 0.11, (0.01, 0.44).

## 4. Discussion

The results extended literature on perspective-taking (Peck et al., 2013; Banakou et al., 2016; Chen et al., 2021c) by comparing the affective and cognitive mediators underlying VRPT’s effect on prejudice. It was found that VRPT of an immigrant outgroup, when compared to VRPT of one’s ingroup, contributes to improved attitudes and reduced negative stereotypes toward the outgroup (supporting H1). Specifically, the effect of VRPT on both attitudes and stereotypes was positively mediated by feelings of empathy toward the outgroup character (supporting H2). Affective appeals are effective in tackling biases rooted in both affect and cognition (Edwards, 1990). Perspective-taking of an outgroup character in distress may trigger the affective experience of experiencing another’s struggles as if it they were one’s own. These findings are in line with previous studies on non-VR perspective-taking. For example, Dovidio et al. (2004) found, among a host of affective and cognitive variables, that parallel empathy—shared feelings of anger and injustice with a victimized target—to be the only significant mediator influencing racial attitudes. They did not test situational attributions as a mediating variable.

To the best of our knowledge, this study provides the first empirical test of empathy and situational attributions as dual mediators of VRPT’s effect on measures of prejudice. In an earlier study that compared cognitive and affective mechanisms underlying VRPT (Chen et al., 2021b), participants were randomly assigned to focus on their own emotions (affective perspective-taking) or thoughts (cognitive perspective-taking) when embodying an ethnic minority avatar in VR. In this case, situational attributions did not mediate the effect of cognitive perspective-taking on attitudes toward the minority outgroup. The present study advances this approach by testing both empathy and situational attributions as potential mediators of the *same* VRPT manipulation. Responding to RQ1, we found that situational attributions did not mediate the effect of outgroup VRPT on either attitudes or stereotypes.

It is possible that the act of perspective-taking, regardless of the group target, was sufficient to “train” a general situational focus when explaining others’ behavior, thus producing minimal differences in attribution levels between the two conditions. The average degree of situational attributions made about outgroup behavior in both the ingroup VRPT (*M* = 5.91, SD = 5.94) and outgroup VRPT conditions (*M* = 5.95, SD = 1.08) was relatively high, more than two scale points higher than the midpoint of 3.5. Perspective-taking may have led to transfer effects, where greater attentiveness to situational cues may have extended from one target group to another (Vezzali and Giovannini, 2012; Lolliot et al., 2013). Indeed, applying thinking patterns or skills learned in one context to another is an active, cognitive process, rather than an automatic one (Wright et al., 2008). Thus, the cognitive processes triggered by perspective-taking may have encouraged a situational attribution style more generally, regardless of target group identity. In contrast, the automatic processes underlying affective empathy may be target-specific. Further research should unpack these divergent affective and cognitive mechanisms, and whether they can be generalized beyond a specific target group (Mado et al., 2021). In response to RQ2, an analysis of pairwise comparisons showed that the indirect effect of empathy on attitudes was significantly greater than that of situational attributions. This study provides further empirical support for the role of affective perspective-taking mechanisms.

Notably, our findings diverge from Vescio et al. (2003) study, which not only found significant mediating effects for both empathy and situational attributions in a non-VR context, but also found situational attributions to carry the stronger effect. These differences may be due to the *medium* of perspective-taking. While non-VR perspective-taking manipulations require participants to actively imagine what another person might be thinking or feeling, VR’s immersive affordances and ability to viscerally showcase affective stimuli may preclude the need for this level of effortful cognition (Shin, 2018). A meta-analysis of 43 experiments found that VR is effective in inducing feelings of compassion or concern for others. However, VR is less effective in inducing the cognitive component—the acknowledgment of another person’s thoughts or feelings (Martingano et al., 2021). While affective empathy can be induced automatically and spontaneously through evocative stimuli (Neumann and Strack, 2000; Yu and Chou, 2018), cognition requires more active attention and mentalizing (Gilovich et al., 2000; Roßnagel, 2000). As such, by providing users with vivid sensory experiences that leave little room for imagination, VRPT may more effectively tap into the automatic processes constituting affective empathy. Additionally, VRPT may effectively “do all the work” for participants—by literally putting participants in the perspective of another, VRPT precludes any cognitive effort. Conversely, providing perspective-takers with a less immersive stimulus—such as a written testimonial or narrative (Vescio et al., 2003; Batson et al., 2016), may require more deliberate cognitive engagement, including shifts in attribution style.

This study offers important theoretical insights into the competing mechanisms of VRPT in shaping prejudice, but it is not without design limitations. The absence of a “true control” where no perspective-taking took place makes it difficult to compare findings with non-VR perspective-taking studies. Future experimental replications should include a non-perspective-taking condition single out latent effects of VRPT on attribution style. Furthermore, while empathy and attributions feature prominently in the literature on perspective-taking and intergroup biases, other affective and cognitive mediators may be considered for a fuller picture (Dovidio et al., 2004). Follow-up studies with a more robust sample size may test the relative indirect effects of other variables such as the well-studied concept of self-other overlap and cognitive empathy. To empirically test the importance of a perspective-taking medium, the direct and indirect effects of outgroup VRPT on intergroup bias should also be directly compared against those of non-VR perspective-taking, where an outgroup viewpoint is imagined.

Although we were sufficiently powered to detect a moderate experimental effect, the lack of power to detect a small effect may have hindered our ability to detect a significant indirect effect of VRPT via a shift in attributions. Our sample was also limited to a student population, and further research should test how this model generalizes to a more diverse sample of participants.

Lastly, we acknowledge that the self-evaluative measures used to capture prejudice are limited due to social desirability concerns, and may not necessarily inform intergroup behaviors and outcomes (Brauer, 2023). Although self-report measures capture specific facets of prejudice and intergroup emotions in the most face-valid manner, future work could triangulate these measures with physiological metrics. One study, for example, measured alertness toward stereotypical vs. non-stereotypical portrayals of different ethnic groups in VR using an electroencephalogram (EEG) (D’Errico et al., 2020). Another aspect that can contribute insights to the field is the nonverbal aspect of participants’ reactions, which were not measured in the current study. Bodily movements and expressions are key signals of emotion (Reed et al., 2020). VR researchers could benefit from coding non-verbal emotion expressions of empathy during VRPT experiences to provide convergent evidence for the influence of VRPT on prosocial emotions. To further understand how emotions are communicated during VR experience, future research should also consider the works on multimodality of communication (Mehu and van der Maaten, 2014). Recording the bodily reactions of the participants in terms of agreement and disagreement can be used to triangulate with self-report measures (Poggi et al., 2011) in order to provide robust research findings.

## Data availability statement

The datasets presented in this article are not readily available because it is restricted by the grantor. Requests to access the datasets should be directed to chenhh@ntu.edu.sg.

## Ethics statement

The studies involving humans were approved by NTU-Institutional Review Board. The studies were conducted in accordance with the local legislation and institutional requirements. The participants provided their written informed consent to participate in this study.

## Author contributions

VC: Conceptualization, Data curation, Funding acquisition, Investigation, Methodology, Project administration, Supervision, Writing–review & editing, Writing–original draft. GI: Methodology, Writing–original draft, Writing–review & editing.

## Funding

The author(s) declare financial support was received for the research, authorship, and/or publication of this article. This research is supported by the Ministry of Education, Singapore, under its Academic Research Fund Tier 1 Grant (MOE2020-T1-001-080/RG41/20).

## Conflict of interest

The authors declare that the research was conducted in the absence of any commercial or financial relationships that could be construed as a potential conflict of interest.

## Publisher’s note

All claims expressed in this article are solely those of the authors and do not necessarily represent those of their affiliated organizations, or those of the publisher, the editors and the reviewers. Any product that may be evaluated in this article, or claim that may be made by its manufacturer, is not guaranteed or endorsed by the publisher.
